# High levels of miR-146a-5p in COVID-19 patients are associated with *Klebsiella* lung coinfection

**DOI:** 10.3389/fimmu.2026.1846431

**Published:** 2026-07-09

**Authors:** Gloria Pérez-Rubio, Ricardo A. Herrera-Sicairos, Leslie Chavez-Galan, Samuel Campista-León, Luz Isela Peinado-Guevara, Ivette Buendia-Roldan, Lucero A. Ramon-Luing, Ingrid Fricke-Galindo, Brandon Bautista-Becerril, Ramcés Falfán-Valencia

**Affiliations:** 1 Pneumogenomics Laboratory, Instituto Nacional de Enfermedades Respiratorias Ismael Cosío Villegas, Mexico, Mexico; 2 Laboratory of Integrative Immunology, Instituto Nacional de Enfermedades Respiratorias Ismael Cosío Villegas, Mexico, Mexico; 3 Laboratory of Microbiology and Applied Biology, Faculty of Biology, Universidad Autónoma de Sinaloa, Culiacán Rosales, Sinaloa, Mexico; 4 Translational Research Laboratory on Aging and Pulmonary Fibrosis, Instituto Nacional de Enfermedades Respiratorias Ismael Cosío Villegas, Mexico, Mexico

**Keywords:** Bacterial coinfection, COVID-19, IMV, Klebsiella, miR-146a-5p

## Abstract

**Background:**

Bacterial coinfections in COVID-19 patients present significant clinical challenges, particularly in severe cases. Previous studies have highlighted the role of microRNAs (miRNAs) in regulating immune responses, with miR-146a-5p and miR-21-5p implicated in inflammation and immune modulation. This study examines the effect of bacterial coinfections on the expression levels of these miRNAs in patients with severe COVID-19.

**Methods:**

We conducted a cohort study involving Mexican mestizo patients ≥18 years hospitalized with severe COVID-19, confirmed via PCR testing. Patients were categorized based on the presence or absence of bacterial coinfection, determined through bronchial aspirate or sputum culture. Blood samples were collected at admission and after 15 days of hospitalization to measure the relative expression levels of miR-146a-5p and miR-21-5p. Additionally, an *in vitro* assay was performed using peripheral blood mononuclear cells (PBMCs) exposed to stimuli (LPS, SARS-CoV-2 Spike Protein, and a combination of both).

**Results:**

Patients with bacterial coinfection were older (43 vs. 41 years, p = 0.046) and had a higher BMI than those without (29.40 vs. 27.51 kg/m2, p = 0.006). The PaO_2_/FiO_2_ ratio was significantly lower in co-infected patients (127.50 vs. 163.75 mmHg, p=0.002), who also exhibited higher rates of severe hypoxemia (35.2% vs. 8.7%), greater need for invasive mechanical ventilation (IMV, 96.0% vs. 34.7, p< 0.001), and higher mortality (80.3 vs. 13.0, p< 0.001). No significant differences in miRNA levels were noted after 15 days of hospitalization, regardless of bacterial presence. The *in vitro* assay did not reveal substantial differences in miRNA expression across stimuli. At the same time, *Klebsiella* spp. was identified as a predominant coinfecting pathogen, with significantly elevated levels of miR-146a-5p observed in these patients (p = 0.017).

**Conclusions:**

Patients with COVID-19 and bacterial coinfection exhibit worse clinical indicators and increased disease severity, demonstrated by older age, higher BMI, severe hypoxemia, greater need for IMV, and increased mortality. Patients with COVID-19 and coinfection with *Klebsiella* spp. showed elevated levels of miR-146a-5p.

## 1 Introduction

Viral respiratory tract infections are associated with high mortality rates. One contributing complication is the potential colonization by other viruses, bacteria, or fungi (1). Coinfection is defined as the detection of additional pathogens in a patient with an infection within 48 hours of hospital admission, whereas superinfection develops 48 hours after the initial infection and hospital admission (2). Bacterial coinfection in COVID-19 has been a widespread concern among healthcare professionals due to overlapping clinical features with bacterial pneumonia. The clinical course of COVID-19 varies significantly; recovery in patients with mild to moderate symptoms can take up to 10 days, whereas in those with critical illness or immunocompromised status, recovery can take approximately 15 days (3). Bacterial coinfection in COVID-19 further complicates this scenario by overlapping with clinical features of bacterial pneumonia. The widespread use of antimicrobial therapy during the pandemic increased antimicrobial resistance, prolonged hospital stays, and increased the risk of morbidity and mortality (4). A meta-analysis revealed that 7% of the hospitalized patients with COVID-19 had a bacterial coinfection, which increased to 14% in intensive care unit (ICU) patients. The most frequently detected bacteria are *Mycoplasma pneumoniae*, *Pseudomonas aeruginosa*, *Haemophilus influenzae*, and *Klebsiella pneumoniae* (5). A study in northern Mexico reported that, in the bronchial secretions of hospitalized patients with COVID-19, *Acinetobacter baumannii*, *Pseudomonas aeruginosa*, *Klebsiella pneumoniae*, and *Stenotrophomonas maltophilia* were predominant (prevalence of 37.2%, 16.9%, 14.0%, and 14.0%, respectively) (6).

In a cohort Caucasian, after adjusting for older age (≥65 years), male sex, pre-admission comorbidities (diabetes, COPD, heart failure or myocardial infarction, renal disease), and 24-h post-admission, coinfection conferred the greatest increased likelihood for in-hospital mortality (OR = 4.05. CI 95%, 2.29–6.97), ICU admission (OR = 4.47. CI 95%, 2.87–7.09), and need for mechanical ventilation (OR = 3.84, CI 95%, 2.21–6.12) (7).

MicroRNAs (miRNAs) are a family of tiny noncoding RNAs that regulate gene expression. Host-induced miRNAs can function as pro- or antiviral factors and contribute to the cytokine storm associated with SARS-CoV-2 infection. A few of these miRNAs may be important regulators of inflammation and the inhibition of SARS-CoV-2 genome expression (8).

The miRNAs identified as potential biomarkers in COVID-19 are miR-21-5p, miR-146a, miR-126-3p, miR-144, and miR-155; miR-21-5p is the only one showing dysregulated expression in SARS-CoV-2 infection (9). High levels of miR-21-5p are positively correlated with IL-6 and creatine phosphokinase (CPK), which are biomarkers of inflammation and myocardial damage (10). In the Caucasian population, miR‐21-5p was elevated in acute COVID‐19 compared with healthy controls and influenza patients (11). Our work group previously reported that, in young, hospitalized patients with COVID-19, increased expression of miR-21-5p was associated with worse outcomes (12).

MiR-146a-5p is of particular interest, given its ability to induce a negative feedback loop that restrains the activation of the NF-κB pathway by targeting TNF receptor-associated factor 6 (TRAF6) and interleukin-1 receptor-associated kinase 1 (IRAK1). COVID-19 patients who did not respond to tocilizumab after the treatment had lower serum levels of miR-146a-5p and experienced adverse outcomes (13). Reduced miR-146a-5p expression in severe COVID-19 patients is associated with increased IL-6 production (10). Increased levels of IL-6 in COVID-19 are strongly correlated with adverse clinical outcomes (14). On the other hand, *in vitro* assays show that macrophages infected with *Mycobacterium tuberculosis* increase miR-21-5p expression, thereby improving mycobacterial survival and decreasing the secretion of inflammatory cytokines, including IL-1β, IL-6, and TNF-α (15).

However, we do not know how bacterial coinfections affect the microRNA levels. Therefore, the study’s objective was to determine whether the levels of miR-21-5p and miR-146a-5p in patients with critical COVID-19 are affected by bacterial co-infection and to evaluate miR-21-5p levels after 15 days of hospitalization. Additionally, we sought to determine whether miRNA levels were altered under experimental conditions, including LPS and spike protein stimulation, using an *in vitro* model that simulates bacterial co-infections. As a secondary objective, we then evaluated miR-21-5p levels in cells from COVID-19 patients after LPS and spike protein stimulation.

## 2 Materials and methods

### 2.1 Study population

We included Mexican mestizos (Mexican mestizo population arises mainly from the mixture of three founder populations; Amerindian, Spaniards, and a smaller proportion of the African population) 18 years old or older, who were admitted to the hospital with a diagnosis of COVID-19 confirmed by SARS-CoV-2 infection via a nasopharyngeal swab PCR test at the Instituto Nacional de Enfermedades Respiratorias Ismael Cosío Villegas (INER) during July to August 2021. The PCR test was part of the hospital’s routine, along with the physician’s assessment; it was determined whether the patient met the criteria for severe COVID-19 and admission to the hospitalization area.

Upon admission to the hospital, bacterial coinfection was confirmed by culture of the bronchial aspirate or sputum and by biochemical tests. The patients enrolled presented with severe COVID-19 because they had dyspnea, a respiratory rate ≥30 breaths per minute, blood oxygen saturation ≤90%, and PaO_2_/FiO_2_ ≤ 300. Patients who voluntarily discharged themselves, whose biological samples did not meet the required quantity and quality criteria, and those diagnosed with cancer or undergoing treatment were excluded to avoid potential confounders in interpreting the results of the miRNAs under study.

Demographic characteristics, clinical data, and bacterial analysis results were recollected from each participant’s electronic medical records upon arrival at the hospital. The study was conducted in full compliance with the Declaration of Helsinki. The institutional ethics committee approved the study protocol (approval number: C53-20, B11-23).

### 2.2 Obtaining blood samples, miRNAs isolation, and quantification

Blood samples were collected in ethylenediaminetetraacetic acid (EDTA) tubes upon arrival and after 15 days of hospital stay. The plasma was separated to evaluate miRNAs. Afterward, RNA extraction was performed with the miRNeasy Serum/Plasma Advanced Kit (Cat. No./ID: 217204, Qiagen, Hilden, Germany). For normalization, synthetic *Caenorhabditis elegans* 39-3p (cel-miR-39-3p) was added as an external reference miRNA (1.6 × 10^8^ copies/μL). The purpose of cel-miR-39-3p in miRNA expression assays is to act as an exogenous spike-in control. It tracks methodological performance and corrects for technical variability during RNA extraction and cDNA synthesis (16). The next step was to quantify the RNA obtained via spectrophotometry at 260 nm using the NanoDrop™ 2000/2000c instrument (Id: ND-2000, Thermo Scientific™, Foster City, CA, USA). Contamination with organic compounds and proteins was established using the ratios of the readings at 260/230 and 260/280, respectively; the samples were considered free of contaminants when the ratios were between 1.7 and 2.0. Then, they were stored at −20 °C until use.

The quality control, extraction of miRNAs, and relative expression of miR-21-5p and miR-146a-5p were described previously (12). We measure relative expression after 15 days of hospital stay because miR-21-5p has previously been associated with worse COVID-19 outcomes.

### 2.3 *In vitro* assay

We randomly selected peripheral blood mononuclear cells from 12 patients with moderate ARDS. PBMCs were isolated from 15 mL of blood using standard Lymphoprep ™ (Accurate Chemical-Scientific, Westbury, NY, USA). The PBMCs were then cryopreserved until use, ensuring their integrity for subsequent experiments by assessing viability using trypan blue dye exclusion. The PBMCs were seeded in 24-well, flat-bottomed cell culture plates (Corning Costar Sigma-Aldrich, St Louis, MO, USA) at a density of 5×10^5^ cells/well in RPMI 1640 medium supplemented with 2 mM L-glutamine, 1 M HEPES (Gibco™, Paisley, Scotland, UK), an antibiotic–antimycotic solution of Penicillin–Streptomycin–Amphotericin B (Gibco™ Paisley, Scotland, UK), and 10% fetal bovine serum (Gibco™ Paisley, Scotland, UK). The cell cultures were maintained for 24 hours at 37 °C in a controlled, humidified atmosphere containing 5% CO_2_ to ensure optimal growth and function. Cells were subject of four conditions: 1) with 1 mg/mL of lipopolysaccharide (LPS catalog #L4516, Sigma-Aldrich, St Louis, MO, USA), 2) with 1 mg/mL of SARS-CoV-2 S1/S2 spike protein (catalog #794206, Biolegend, San Diego, CA, USA), 3) LPS + Spike, and 4) untreated (only culture media); we follow conditions described previously to stimulate us the cells (17). We measure the relative expression in the supernatant of the stimulated culture, as previously described (12).

### 2.4 Statistical analysis

The normality of the variables was assessed using the Shapiro-Wilk test; for quantitative variables with non-normal distributions, the median and interquartile range were reported. For variables with a normal distribution, we used the mean and standard deviation. Categorical variables were presented as percentages, and the exact Fisher’s test was used. A Mann–Whitney U test was employed to analyze miRNA expression levels between the groups. All statistical analyses were performed with SPSS 21.0 software.

## 3 Results

### 3.1 Clinical characteristics and prevalence of coinfections in hospitalized patients with COVID-19

Ninety-seven hospitalized patients were included, with a median age of 43 years (IQR: 36–51) and a higher prevalence of men (75.26%). The median body mass index (BMI) was 29.22 kg/m² (IQR: 25.21–32.95), suggesting a high prevalence of overweight and obesity in the population (**Supplementary Table 1**).

The median time from symptom onset to hospitalization was 8 days (IQR: 7–11). Regarding respiratory function, the PaO_2_/FiO_2_ ratio was 156 (IQR: 102–210), indicating significant respiratory impairment. Based on ARDS severity, 30.9% of patients had mild, 46.4% moderate, and 22.7% severe ARDS.

About comorbidities, 25.77% of patients had type 2 diabetes mellitus (T2DM), 22.68% had systemic arterial hypertension (SAH), and almost a third of the patients (32.99%) were current smokers. Furthermore, 69.07% of patients required invasive mechanical ventilation (IMV), with a median duration of 20 days (IQR: 13–29) on IMV. The overall mortality rate among all admitted patients was 32.99% (n = 97).

Regarding bacterial coinfections, 52.58% of patients had respiratory tract coinfections, 16.49% had urinary tract coinfections, and 13.4% had positive blood cultures, which underlines the high prevalence of coinfections in these patients (**Supplementary Figure 1**).

### 3.2 Comparison of clinical characteristics in patients with and without bacterial coinfection in the respiratory tract

The clinical features of two groups of hospitalized COVID-19 patients were compared: one with respiratory tract bacterial coinfection (n = 51) and the other without coinfection (n = 46) (**Table 1**).

**Table 1:** Demographic and clinical data of patients with COVID-19.

Variable	Respiratory tract bacterial coinfection		p-value
	Positive n=51	Negative n=46	
Age, years	43 (39-52)	41 (31-41)	**0.046**
Male, n (%)	41 (80.3)	32 (69.5)	0.159
BMI, kg/m^2^	29.4 (27.2-35.1)	27.5 (24.4-30.4)	**0.006**
Symptoms onset (days)	9 (7-12)	8 (6-10)	0.052
PaO_2_/FiO_2_ (mmHg)	127.5 (74.5-195.0)	163.7 (129.6-232.0)	**0.002**
Classification PaO_2_/FiO_2_ (mm Hg), n (%)			
Mild	11 (21.5)	19 (41.3)	
Moderate	22 (43.1)	23 (50.0)	**0.001**
Severe	18 (35.2)	4 (8.7)	
Comorbidities, n (%)			
T2DM	12 (23.5)	13 (28.2)	0.381
SAH	13 (25.4)	9 (19.5)	0.326
CD	0	2 (4.3)	0.461
RD	1 (1.9)	3 (6.5)	0.270
TS	18 (35.2)	14 (30.4)	0.385
IMV, n (%)	49 (96.0)	16 (34.7)	**< 0.001**
Length IMV, days	23 (15-34)	0 (0-9)	**< 0.001**
Deceased, n (%)	41 (80.3)	6 (13.0)	**< 0.001**

Continuous data are presented as medians with interquartile ranges (IQRs), and categorical data are presented as counts and percentages. BMI, body mass index; T2DM, Type 2 diabetes mellitus; SAH, systemic arterial hypertension; CD, Cardiovascular disease; RD, Preexisting chronic respiratory disease; TS, tobacco smoking; IMV, invasive mechanical ventilation. The p-value was calculated using the Mann–Whitney U or Fisher’s exact test.

Text in bold shows statistically significant p-values.

Patients in the group with bacterial coinfection were older than those without coinfection (43 *vs*. 41 years, p = 0.046). BMI was also significantly higher in the group with respiratory bacterial infections (29.40 *vs*. 27.51 kg/m^2^, p = 0.006). The PaO_2_/FiO_2_ ratio, a critical indicator of lung function and oxygenation, was notably lower (p = 0.002) in patients with respiratory bacterial coinfections (127.50 mmHg) compared to those without coinfections (163.75 mmHg). The PaO_2_/FiO_2_ classification showed that patients with respiratory bacterial coinfection had a higher proportion of severe hypoxemia (35.2% vs. 8.7%) than those without bacterial coinfection (**Table 1**).

Regarding comorbidities, no statistically significant differences were observed between the two groups. The need for IMV was higher among patients with respiratory tract bacterial coinfections than among those without (96.08% vs. 34.78%, p < 0.001). Furthermore, IMV duration was longer (p < 0.001) in the group with respiratory coinfections (median 23 days) than in the group without coinfections. Mortality was higher (p<0.001) in patients with respiratory coinfections (80.3%) compared to those without infections (13.0%), as shown in **Table 1**.

**Figure 1:**
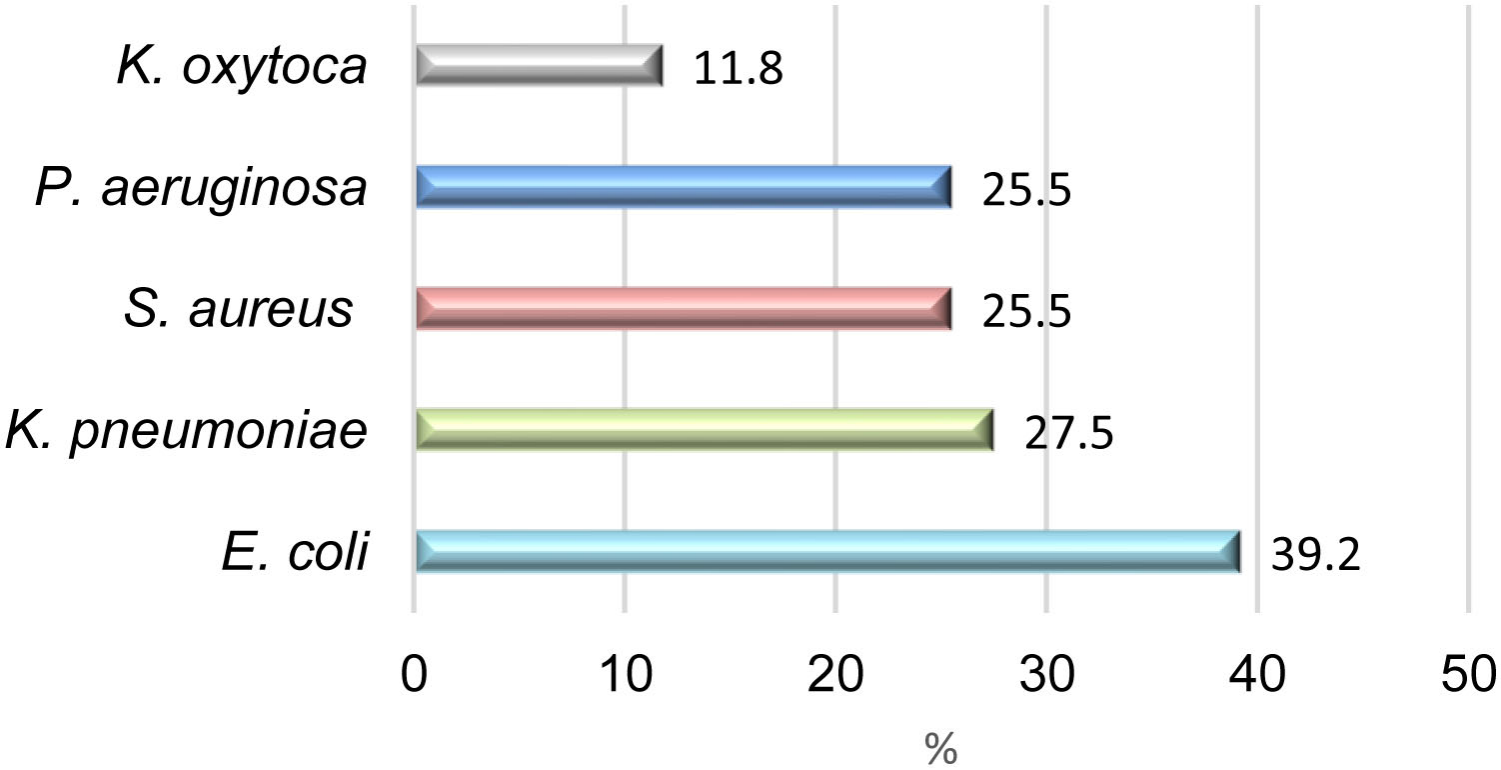
The most frequent bacterial species found in the present study. We showed the top five.

We analyzed 51 patients with positive bacterial culture results and bacterial composition. The predominant phyla were Pseudomonadota (94.1%) and Bacillota (27.5%). At the family level, 74.5% of patients were infected with Enterobacteriaceae. The top five genera were Enterobacter (74.5%), Klebsiella (45.0%), Escherichia (39.2%), Pseudomonas (27.5%, and Staphylococcus (25.5%). The top five species were *Escherichia coli* (39.2%), *Klebsiella pneumoniae* (27.5%), *Staphylococcus* aureus (25.5%), *Pseudomonas aeruginosa* (25.5%), and *Klebsiella oxytoca* (11.8%) (**Figure 1**).

We compared the fold-change of miR-21-5p and miR-146a-5p upon admission to the hospital and after 15 days of stay in the hospital according to the presence or absence of phyla, family, or genus previously described (**Supplementary Table 2**). We found significant differences in miR-146a-5p between patients co-infected with *Klebsiella* spp. and those with other coinfecting bacteria (p = 0.017), with levels 3.25 times higher in patients with *Klebsiella* spp. (**Table 2**).

**Table 2:** Fold-change of miRNAs in patients with COVID-19 co-infected with *Klebsiella spp* versus other bacteria.

Fold change	Positive *Klebsiella sp*p (n=23)	Negative *Klebsiella sp*p (n=28)	p
miR-21-5p	0.640 (0.250-1.820)	0.615 (0.255-1.280)	0.899
miR-146a-5p	0.960 (0.320-2.310)	0.295 (0.127-0.762)	**0.017**

Showing median and percentiles 25 and 75. The p-value was calculated using the Mann–Whitney U test.

Text in bold shows statistically significant p-values.

It should be noted that these patients showed no statistically significant differences in age, sex, BMI, PaO_2_/FiO_2_, and IMV. Although IMV duration and mortality rates did not differ, COVID-19 patients with Klebsiella coinfection required more days of life support (23 days vs. 18 days in those without Klebsiella). A similar trend was seen in mortality rates, with 60% of coinfected patients dying versus 42.8% of those without the coinfection (**Table 3**). This suggests that the changes in miRNA are likely related to the presence of *Klebsiella* spp.

**Table 3:** Demographic and clinical data of patients with COVID-19 with coinfection by *Klebsiella* spp.

Variable	Positive *Klebsiella* spp. (n=23)	Negative *Klebsiella* spp. (n=28)	p-value
Age (years)	48 (42-52)	42 (33-52)	0.069
Male, n (%)	17 (73.9)	24 (85.7)	0.241
BMI (kg/m^2^)	29.3 (27.0-35.5)	29.7 (27.4-34.9)	0.899
PaO_2_/FiO_2_ (mmHg)	128 (70-225)	126 (75-173)	0.899
IMV, n (%)	22 (95.6)	27 (96.4)	0.703
Days of IMV	23 (20-38)	18 (13-28)	0.943
Mortality, n (%)	14 (60.8)	12 (42.8)	0.159

BMI, Body mass index; PaO_2_/FiO_2,_ Ratio of the partial pressure of oxygen/fraction of inspired oxygen; IMV, Invasive mechanical ventilation. We show the median (IQR), except for male, ARDS, IMV, and mortality.

We recovered samples from patients who were still hospitalized 15 days after their admission, with 11 positive *Klebsiella* spp. and 28 negatives. However, no differences were observed in this follow-up for miR-21-5p nor miR-146a-5p expression (p>0.05). The same analysis was performed for *Klebsiella* species (*K. pneumoniae, K. oxytoca, and K. aerogenes*), and no significant differences were observed (**Supplementary Table 3**).

**Figure 2:**
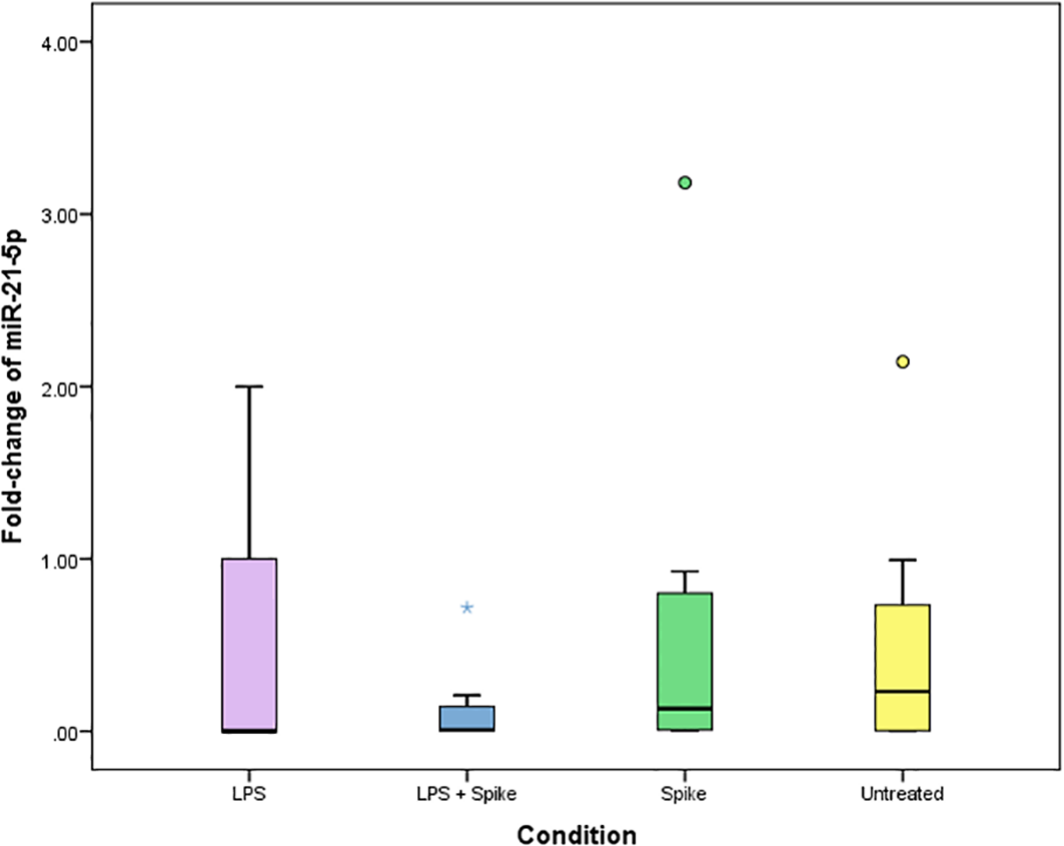
*In vitro* assay of supernatant of a culture of peripheral blood mononuclear cells in a subgroup of patients in different conditions, p = 0.424 by the Kruskal-Wallis test.

The *in vitro* assay was performed with the 12 samples of patients with moderate ARDS (**Supplementary Table 4**) in duplicate under each condition; there were no statistically significant differences in fold-change for miR-21-5p between the stimulus conditions LPS, LPS + Spike, or Spike (p = 0.424) (**Figure 2**) (**Supplementary Table 5**).

## Discussion

The impact of bacterial coinfections on COVID-19 outcomes has been a significant area of investigation. Studies have consistently shown that bacterial coinfections in COVID-19 patients are associated with severe clinical manifestations and worse outcomes. Hospital-acquired bacterial infections among ICU-admitted COVID-19 patients increased hospitalization length and mortality (18). Bacterial coinfections significantly increase the likelihood of adverse outcomes in COVID-19 patients as they are associated with a higher in-hospital mortality rate (38%), a higher risk of ICU admission (59%), and a higher need for mechanical ventilation (80%) (19). Our study population of patients with COVID-19 and bacterial coinfection was older than those without coinfection, had a higher BMI, showed a greater proportion of severe hypoxemia, experienced more days of IMV, and exhibited higher mortality.

In 2019, 13.7 million infection-related deaths were recorded, of which 7.7 million were related to 33 bacterial pathogens. The top five pathogens were *Staphylococcus aureus, Escherichia coli, Streptococcus pneumoniae, Klebsiella pneumoniae*, and *Pseudomonas aeruginosa* (20). Our results are similar to those reported in Caucasian and Asian COVID-19 patients coinfected by *Escherichia, Klebsiella, Staphylococcus*, and *Pseudomonas* (21–24). Among the most frequent bacteria, we find *Enterobacter and Klebsiella*; both acquire various resistance determinants under the selection pressure of antibiotics widely used during the COVID-19 pandemic. *K. pneumoniae* and *E. cloacae* are suitable plasmid vectors containing resistance genes to β-lactam and non-β-lactam antibiotics (25). In the Caucasian population of COVID-19, patients with carbapenemase-producing *Enterobacterales* infections are more likely to die than patients without coinfection (26). The relative incidence of carbapenem-resistant *Klebsiella pneumoniae* increased by 4.8 times during the COVID-19 period (27).

*Pseudomonas* is a pathogen that causes fatal infections in immunocompromised patients. *P. aeruginosa* can colonize and evolve in SARS-CoV-2-infected environments by altering the production of alginate, which might enhance biofilm formation, increase antimicrobial resistance, and promote superinfection with other pathogens such as *Staphylococcus aureus* (28). In the Caucasian population, *Escherichia* is an opportunistic pathogen that causes secondary bacterial infections in patients with COVID-19. The principal problem is that these bacteria show resistance to ampicillin, cefixime, cefepime, amoxicillin-clavulanic acid, cefuroxime, and ceftriaxone (29).

Lung tissue injury from SARS-CoV-2 infection, as well as bacterial evasion of the immune defense, results in airway dysfunction, cytopathology, tissue destruction, and damage to the protective mucosa in the lung, exacerbating disease severity and increasing the risk of septicemia and admission to the intensive care unit (30).

MiRNAs play a crucial role in regulating host immune responses and have been implicated in the pathogenesis of COVID-19. Low levels of miR-146a-5p in serum were associated with poor responses to tocilizumab and adverse outcomes in COVID-19 patients (31).

The miR-21 gene is abundantly expressed in multiple mammalian cell types and is located on chromosome 17q23.2, downstream of the 3´- untranslated region of the VMP1 (vacuole membrane protein 1) gene (32). miR-21 as a critical switch in immune circuits, controlling the balance between initial pro-inflammatory and later immunoregulatory and anti-inflammatory responses, dysregulation of which contributes to the pathogenesis of inflammatory diseases and infection (33). miR-21 enhances the production of the anti-inflammatory cytokine IL-10 while decreasing the pro-inflammatory activity of NF-κB (34). Previously, we reported that increased levels of miR-21-5p have been linked to severe disease and poor prognosis in young, hospitalized COVID-19 patients (12). In a Caucasian COVID-19 cohort, miR-21-5p levels showed positive correlations with serum IL-6, creatine phosphokinase, inflammation, and myocardial damage (35).

In the present report, miR-21-5p and miR-146a-5p levels are not affected after 15 days of hospital stay and do not present significant differences, regardless of the bacterial species present. The miR-146 family includes miR-146a and miR-146b, found on human chromosomes 5 and 10, respectively. The miR-146a gene resides within the *MIR3142HG* gene (chromosome 5q33.3), while *MIR146B* is in an intergenic region of human chromosome 10 (10q24.32) (36).

MiR-146a-5p decreases the expression of key proteins involved in the canonical NF-κB pathway, including toll-like receptor 4 (TLR-4), myeloid differentiation primary response gene 88 (MyD88), receptor-associated kinase 1. interleukin-1 (IRAK1), and TNF receptor-associated factor 6 (TRAF6) (37). MiR-146a-5p also targets RelB, a key factor in the non-canonical NF-κB pathway, in macrophages and lung fibroblasts (38, 39). Notably, miR-146a-5p is overexpressed in the mitochondria of aging human endothelial cells. In this context, it reduces the expression of Bcl-2 family members, promotes the opening of the permeability transition pore, activates caspases 1 and 3, and influences sensitivity to apoptosis and autophagy, thereby affecting cell function and inflammation (40).

MiR-146a-5p is upregulated by coxsackievirus B and hepatitis C and B viruses, as well as pathogen-related molecules such as endotoxins (41). *In vitro* assays revealed that overexpression of miR146a-5p promotes hepatitis C virus assembly and facilitates metabolic pathways that benefit viral infections (42). Our findings suggest that miR146a-5p may play an important role in regulating the inflammatory response in COVID-19 patients coinfected with *Klebsiella*, potentially contributing to a worse prognosis. Previous studies have also shown elevated levels of miR-146a-5p in immunocompetent cells following various bacterial infections, including *Mycobacterial* species, enteric *Salmonella*, and *Helicobacter* (43–45).

MiR-146a-5p was the first NF-κB-dependent miRNA identified, upregulated by various immune mediators, such as LPS, IL-1β, and TNF-α (30). miR-146a-5p overexpression can be induced by pro-inflammatory conditions and by replicative or stress-induced senescence, reinforcing the hypothesis of miR-146 as a critical role in inflammation (46). The extracellular vesicles of alveolar macrophages infected with methicillin-resistant *Staphylococcus aureus* deliver miR-146a-5p and TNF-α, inducing necroptosis (47).

In the *in vitro* assay, we expected to observe increased miR-21-5p expression in cells stimulated with LPS and LPS + spike protein; however, no changes were observed with either stimulus. Notably, the cells used in this assay were derived from patients with COVID-19 and moderate ARDS. These patients likely exhibited immune dysregulation, similar to that observed in patients with persistent COVID-19, in which chronic T-cell activation not only contributes to inflammation but also leads to T-cell exhaustion (48).

Our study is not without limitations, including its single-center design and reliance on retrospective medical record data, which may be incomplete. The modest sample size is another limitation: when analyzing miRNA levels by species, the number of subjects included per group was small due to the high bacterial diversity. 43% of patients had coinfection with 2 or more bacteria, a condition that is complex to characterize. For samples collected after 15 days of hospitalization, treatments may have affected miRNA levels. The *in vitro* assay was again limited by the small sample size.

Nevertheless, we demonstrated that patients with severe COVID-19 and *Klebsiella* spp. coinfection had higher levels of miR-146a-5p. This study also underscores the importance of microRNAs in infectious diseases and their progression.

## Conclusion

Patients with COVID-19 and bacterial coinfection show worse clinical indicators and greater disease severity, evidenced by older age, high BMI, severe hypoxemia, greater need for IMV, as well as higher mortality. Patients with COVID-19 and coinfection with *Klebsiella* spp. showed high levels of miR-146a-5p.

## Data Availability

The original contributions presented in the study are included in the article/**Supplementary Material**. Further inquiries can be directed to the corresponding author.
