# Supplementary material for: High levels of miR-146a-5p in COVID-19 patients are associated with Klebsiella lung coinfection
Source: Front Immunol. 2026 Jul 9;17:1846431. doi: 10.3389/fimmu.2026.1846431 (PMC13391917; doi:10.3389/fimmu.2026.1846431)

Supplementary Material

# Supplementary Tables

# Supplementary Table 1. Demographic and clinical features of hospitalized COVID-19 patients.

| Characteristic | n = 97 |
| --- | --- |
| Age, years | 43 (36-51) |
| Male sex (n, %) | 73 (75.26%) |
| BMI, kg/m^2^ | 29.22 (25.21-32.95) |
| Symptom onset, days | 8 (7-11) |
| PaO_2_/FiO_2_ | 156 (102-210) |
| ARDS severity (n, %) |  |
| Mild | 30 (30.93%) |
| Moderate | 45 (46.39%) |
| Severe | 22 (22.68%) |
| Comorbidities (n, %) |  |
| T2DM | 25 (25.77%) |
| SAH | 22 (22.68%) |
| Cardiovascular diseases | 2 (2.06%) |
| Chronic respiratory disease (preexisting) | 4 (4.12%) |
| Current smokers (n, %) | 32 (32.99%) |
| IMV (n, %) | 65 (69.07%) |
| Days requiring IMV | 20 (13-29) |
| Deceased | 32 (32.99%) |
| Bacterial coinfections (n, %) |  |
| Respiratory tract | 51 (52.58%) |
| Urine cultures | 16 (16.49%) |
| Blood cultures | 13 (13.4%) |

# BMI: Body mass index; PaO_2_/FiO_2_: Ratio of the partial pressure of oxygen/fraction of inspired oxygen; T2DM: Type 2 diabetes mellitus; SAH: Systemic arterial hypertension; IMV: Invasive mechanical ventilation. We show the median (IQR), except for male, ARDS, comorbidities, current smokers, IMV, deceased, and bacterial coinfection.

# Supplementary Table 2. Fold-change of miRNAs evaluated in patients with COVID-19 with bacterial coinfection and without bacterial coinfection.

| Fold-change | Pseudomonadota (positive) (n=48) | Pseudomonadota (negative) (n=18) | p-value |
| --- | --- | --- | --- |
| miR-21-5p | 0.66 (0.27-1.28) | 0.15 (0.11-0.99) | 0.972 |
| miR-146a-5p | 0.49 (0.22-2.07) | 0.23 (0.13-0.33) | 0.248 |
| miR-21-5p_15days* | 0.74 (0.17-3.31) | 0.05 (0.04-0.06) | 0.480 |
|  | Bacillota (positive) (n=14) | Bacillota (negative) (n=52) |  |
| miR-21-5p | 0.41 (0.14-1.60) | 0.72 (0.26-1.28) | 0.392 |
| miR-146a-5p | 0.41 (0.15-1.53) | 0.72 (0.27-1.27) | 0.392 |
| miR-21-5p_15days* | 0.90 (0.03-3.29) | 0.59 (0.17-5.18) | 1.000 |
|  | Enterobacteriaceae (positive) (n=38) | Enterobacteriaceae (negative) (n=28) |  |
| miR-21-5p | 0.59 (0.27-1.28) | 0.72 (0.19-1.68) | 0.469 |
| miR-146a-5p | 0.57 (0.23-2.13) | 0.30 (0.09-0.80) | 0.575 |
| miR-21-5p_15days* | 0.61 (0.19-5.29) | 0.44 (0.05-2.56) | 1.000 |
|  | *Enterobacter* (positive) (n=9) | *Enterobacter* (negative) (n=57) |  |
| miR-21-5p | 0.64 (0.36-1.55) | 0.61 (0.25-1.35) | 0.948 |
| miR-146a-5p | 1.72 (0.35-2.78) | 0.35 (0.19-1.53) | 0.424 |
| miR-21-5p_15days* | 0.59 (0.14-3.33) | 0.55 (0.07-3.30) | 1.000 |
|  | *Escherichia* (positive) (n=20) | *Escherichia* (negative) (n=46) |  |
| miR-21-5p | 0.90 (0.43-2.10) | 0.63 (0.23-1.25) | 0.690 |
| miR-146a-5p | 0.34 (0.20-3.53) | 0.46 (0.21-1.38) | 0.862 |
| miR-21-5p_15days* | 0.46 (0.25-3.90) | 0.72 (0.07-3.31) | 1.000 |
|  | *Pseudomonas* (positive) (n=14) | *Pseudomonas* (negative) (n=52) |  |
| miR-21-5p | 0.61 (0.20-1.25) | 0.64 (0.27-1.67) | 0.820 |
| miR-146a-5p | 0.40 (0.13-2.95) | 0.43 (0.23-2.07) | 0.820 |
| miR-21-5p_15days* | 0.46 (0.17-3.27) | 0.72 (0.06-3.36) | 1.000 |
|  | *Staphylococcus* (positive) (n=13) | *Staphylococcus* (negative) (n=53) |  |
| miR-21-5p | 0.39 (0.14-1.68) | 0.79 (0.26-1.28) | 0.250 |
| miR-146a-5p | 0.30 (0.15-0.77) | 0.60 (0.20-2.19) | 0.187 |
| miR-21-5p_15days* | 0.90 (0.03-3.29) | 0.59 (0.17-5.18) | 1.000 |

# Shown median and percentiles 25 and 75. *We recovered a sample 15 days after the hospital stay of 39 patients

# Supplementary Table 3. Change in miRNA expression evaluated in patients with COVID-19, with and without bacterial coinfection at the species level.

| Fold-change | *Klebsiella pneumoniae* (+) | *Klebsiella pneumoniae* (-) | p-value |
| --- | --- | --- | --- |
| miR-146a-5p | 1.0 (0.0-3.0) | 0.5 (0.0-3.2) | 0.991 |
| miR-21-5p | 1.0 (0.0-2.0) | 0.5 (0.0-1.2) | 0.309 |
| miR-21-5p_15days* | 0.0 (0.0-3.0) | 1.0 (0.0-5.2) | 0.549 |
|  | *Klebsiella aerogenes* (+) | *Klebsiella aerogenes* (-) |  |
| miR-146a-5p | 0.49 (0.22-2.07) | 0.23 (0.13-0.33) | 0.248 |
| miR-21-5p | 0.41 (0.14-1.60) | 0.72 (0.26-1.28) | 0.392 |
| miR-21-5p_15days* | 0.41 (0.14-1.60) | 0.72 (0.26-1.28) | 0.392 |
|  | *Klebsiella oxytoca* (+) | *Klebsiella oxytoca* (-) |  |
| miR-146a-5p | 5.0 (3.5-5.0) | 0.0 (0.0-2.2) | 0.470 |
| miR-21-5p | 2.0 (1.5-2.5) | 0.0 (0.0-1.0) | 0.310 |
| miR-21-5p_15days* | 2.0 (1.0-4.0) | 1.0 (0.0-3.0) | 0.847 |

# Shown median and percentiles 25 and 75. *We recovered samples 15 days after the hospital stay of 39 patients.

# Supplementary Table 4. Characteristics of patients included in the *in vitro* assay.

| Characteristics | n = 12 |
| --- | --- |
| Age, years | 57 (51-67) |
| Male sex (n, %) | 8 (66.6) |
| BMI, kg/m^2^ | 30 (27-38) |
| PaO_2_/FiO_2_ | 149 (135-160) |
| IMV (n, %) | 65 (69.07%) |
| Days requiring IMV | 12 (1-15) |
| Deceased (n, %) | 6 (50) |

# BMI: Body mass index; PaO_2_/FiO_2_: Ratio of the partial pressure of oxygen/fraction of inspired oxygen; IMV: Invasive mechanical ventilation. We show the median (IQR), except for male, IMV, and deceased.

# Supplementary Table 5. *In vitro* assay of supernatant of a culture of peripheral blood mononuclear cells in a subgroup of patients.

| Condition | Fold-Change of miR-21-5p |
| --- | --- |
| LPS | 1.0E-07 (1.0E-07-1.000) |
| LPS+Spike | 0.006 (0.004-0.178) |
| Spike | 0.130 (0.007-0.832) |
| Control | 0.229 (0.003-0.962) |

# Shown median and percentiles 25 and 75.

## Supplementary Figure

**Supplementary Figure 1. Bacterial coinfections in the population.**


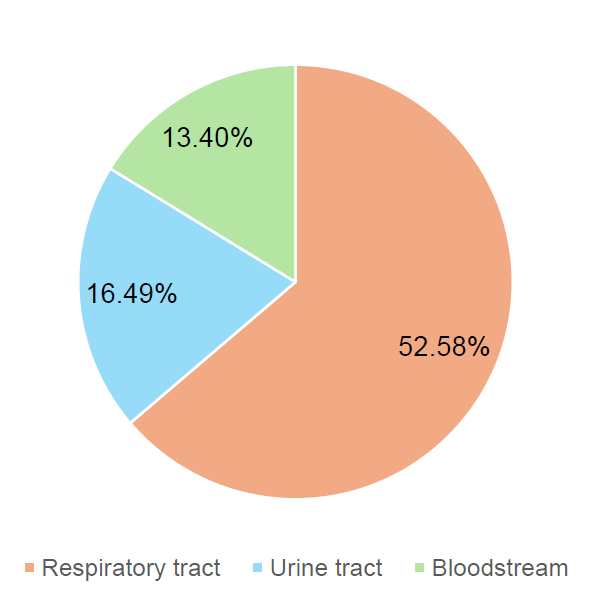

Supplement: Supplementary file 1 [file Table1.docx]
